# Regulatory Non-Coding RNAs in Pluripotent Stem Cells

**DOI:** 10.3390/ijms140714346

**Published:** 2013-07-11

**Authors:** Alessandro Rosa, Ali H. Brivanlou

**Affiliations:** 1 Department of Biology and Biotechnology “Charles Darwin”, Sapienza University of Rome, Rome 00185, Italy; E-Mail: alessandro.rosa@uniroma1.it; 2 Laboratory of Molecular Vertebrate Embryology, The Rockefeller University, New York, NY 10065, USA

**Keywords:** embryonic stem cells, induced Pluripotent Stem Cells, microRNA, long non-coding RNA, pluripotency, reprogramming

## Abstract

The most part of our genome encodes for RNA transcripts are never translated into proteins. These include families of RNA molecules with a regulatory function, which can be arbitrarily subdivided in short (less than 200 nucleotides) and long non-coding RNAs (ncRNAs). MicroRNAs, which act post-transcriptionally to repress the function of target mRNAs, belong to the first group. Included in the second group are multi-exonic and polyadenylated long ncRNAs (lncRNAs), localized either in the nucleus, where they can associate with chromatin remodeling complexes to regulate transcription, or in the cytoplasm, acting as post-transcriptional regulators. Pluripotent stem cells, such as embryonic stem cells (ESCs) or induced pluripotent stem cells (iPSCs), represent useful systems for modeling normal development and human diseases, as well as promising tools for regenerative medicine. To fully explore their potential, however, a deep understanding of the molecular basis of stemness is crucial. In recent years, increasing evidence of the importance of regulation by ncRNAs in pluripotent cells is accumulating. In this review, we will discuss recent findings pointing to multiple roles played by regulatory ncRNAs in ESC and iPSCs, where they act in concert with signaling pathways, transcriptional regulatory circuitries and epigenetic factors to modulate the balance between pluripotency and differentiation.

## 1. Introduction

Pluripotency is defined, in a broad sense, as the ability of a cell to give rise to derivatives of the three germ layers, ectoderm, mesoderm and endoderm, and the germ line. In mammals, this property is restricted to embryonic cells until the stage of blastocyst, in which a small number of cells that constitute the Inner Cell Mass (ICM) are still pluripotent. Such pluripotent cells exist very transiently during embryonic development. Upon gastrulation and during subsequent embryonic development, they lose pluripotency and progressively acquire a specialized character. Embryonic stem cells (ESCs) can be derived from the ICM of human and mouse blastocysts; are pluripotent and can self-renew *in vitro* indefinitely.

ESC pluripotency is tightly regulated. Amongst several signaling pathways, the TGF-β pathway has been shown to play a central role [1]. Interestingly, the two branches of the pathway play different roles in human and in mouse. More precisely, in human ESCs (hESCs) the Nodal/Activin branch is both necessary and sufficient to sustain pluripotency [2,3], whereas in mouse ESC (mESCs) the bone morphogenetic protein (BMP) branch is required for maintaining self-renewal and prevent differentiation [4]. Downstream of signaling pathways, the maintenance of ESCs pluripotency is ensured by a regulatory circuitry including three main core transcription factors (TFs), Oct4, Sox2 and Nanog [5–7]. The three core TFs co-occupy a conspicuous set of target promoters and have a dual role. They can activate transcription of genes involved in the maintenance of pluripotency, including their own genes. At the same time, in association with Polycomb Repressive Complexes (PRC1 and PRC2), they silence a subset of lineage-specific genes that play a role in development [8,9]. In ESCs, the promoters of these genes present peculiar bivalent chromatin domains, in which epigenetic histone modifications normally associated with silent genes co-exist with marks of active transcription [10]. Such unique epigenetic profiles are required to keep key developmental genes “poised” in a repressed state that can be quickly turned on.

Pluripotent cells exist in two different states, defined as naïve and primed [11]. Mouse ESCs are considered to be in a naïve ground state of pluripotency that corresponds to the preimplantation epiblast. *In vitro*, they require Leukemia Inhibitory Factor (LIF) and BMP signaling for self-renewal and differentiate in presence of basic Fibroblast Growth Factor (bFGF). They express a typical set of markers and female cells have two active X chromosomes. Functionally, they are able to form chimeric embryos upon injection in a recipient blastocyst. Conversely, mouse Epiblast Stem Cells (EpiSCs) are in a primed state, which corresponds to the post-implantation epiblast. EpiSCs require bFGF and Nodal/Activin signaling for self-renew, present X chromosome inactivation in female cells, and fulfill some criteria for pluripotency, such as the ability to form teratomas, but cannot generate chimeric mice. There is some plasticity in pluripotent cells, as mESCs can be induced to differentiate into EpiSCs by culturing in presence of Activin and bFGF and EpiSCs can be converted to the ground state by transfection with Klf4 or Nanog and culturing in the presence of LIF and BMP [12,13]. Human ESCs share many features with EpiSCs but differ from mESCs in terms of morphology, culture requirements, expression markers and X chromosome inactivation. This evidence led to the hypothesis that hESCs are in a primed state of pluripotency [14].

The seminal work by Shinya Yamanaka showed that pluripotency could be induced in mouse somatic cells by ectopic expression of a defined set of reprogramming factors (RFs) [15]. Since then, reprogramming of somatic cells into induced Pluripotent Stem Cells (iPSCs) has been achieved in human as well [16,17]. Alternative sets of RFs that contain well-known pluripotency factors can be used. The most common are Oct4, Sox2, Klf4 and c-Myc (Yamanaka RFs) and Oct4, Sox2, Nanog and Lin28 (Thomson RFs). iPSCs share with ESCs the pluripotency, meaning that they could virtually be differentiated *in vitro* into all adult cell types. Moreover, they can be derived from human patients as patient-specific iPSCs (PS-iPSCs) that hold the same disease-causing genetic alteration [18]. The mechanisms underlying reprogramming have been deeply investigated and involve a profound change in cell identity. During reprogramming, the epigenetic landscape of the somatic cell of origin shifts to a state proper of the embryonic stem cell, including erasure of repressive marks on the chromatin of pluripotency genes and establishing of bivalent domains on lineage-specific genes [19].

The ability to differentiate into multiple tissues makes ESCs and iPSCs promising tools for regenerative medicine and cell-replacement therapy approaches [20]. However, to fully exploit their potential, the molecular basis of pluripotency must be deeply characterized. Non-coding RNA (ncRNA) molecules, previously regarded to exert only passive roles in the cell, are conversely primary players to define the cell identity. Rather than the coding portion of the genome, it is now clear that its non-coding counterpart is correlated with the greater complexity of higher eukaryotes [21]. Recently, ncRNAs are also emerging as new regulatory factors in pluripotent cells. Among small non-coding RNAs (<200 nucleotides), microRNAs (miRNAs) are now considered major regulators of development, metabolism, differentiation and homeostasis in all multicellular organisms [22–26]. miRNAs are also involved in several human diseases, including cancer [27]. Biogenesis of miRNAs requires a multistep process [23]. miRNAs are generally transcribed by RNA polymerase II as part of introns of mRNA genes, or from intergenic regions. The miRNA primary precursor (pri-miRNA) is then processed in the nucleus by the Microprocessor complex [28,29], comprised of the cleavage enzymes Drosha, DGCR8 and other factors, releasing a stem-loop precursor (pre-miRNA). The pre-miRNA is then exported in the cytoplasm and cleaved by the RNAse III enzyme Dicer, which is also involved in the maturation of short interfering RNAs (siRNAs) [30,31]. The mature miRNA is finally incorporated as a single strand in the RNA Induced Silencing Complex (RISC) [32]. Guided by the miRNA, RISC binds the 3′UTR and/or the coding sequences of target mRNAs resulting in inhibition of translation and/or degradation. A single miRNA can inhibit several targets and a single mRNA can be targeted by multiple miRNAs in a combinatorial way [33]. For target recognition by miRNAs, base pairing of a short “seed” sequence, located at the 5′end of the mature miRNA, is required [34]. Families of miRNAs comprise members with identical seed sequences and all miRNAs belonging to a family are thought to share the same targets. Multiple miRNAs can be excised from a single, multicistronic, pri-miRNA transcript. Such clusters can comprise multiple members of a miRNA family as well as unrelated miRNAs.

Despite lacking an open reading frame (ORF), long (>200 nucleotides) non-coding RNAs (lncRNAs) are transcribed by RNA polymerase II and have a structure that resemble protein-coding mRNAs. They are generally spliced from multi-exonic precursors, have a 5′cap and are polyadenylated [35,36]. Some lncRNAs are transcribed from loci which overlap or are very close to protein-coding genes, while others are intergenic (long intergenic non-coding RNAs, lincRNAs). Globally, lncRNA expression levels are only slightly lower than protein-coding transcripts [37,38]. Similar to protein-coding and miRNA genes, and differently from other structural non-coding RNAs such as ribosomal RNAs, the expression of lncRNAs is characterized by tissue specificity, and is dynamically regulated during differentiation [39–42]. Moreover, many lncRNA are conserved in terms of sequence and predicted secondary structure [37,38,43]. All together, these elements suggest that lncRNAs may represent a new, still largely unexplored class of functional molecules, potentially involved in multiple biological processes [44–46]. In the last years, the continuous development of powerful sequencing technologies and bioinformatics tools boosted the discovery of thousands novel lncRNAs. According to some estimates, the total number of human lincRNAs would be around 4500 [47]. So far, however, a function has been only assigned to a limited number of lncRNAs. Intracellular localization is often used as a predictive element to get insights into lncRNA molecular mechanisms [48]. Nuclear lncRNAs can regulate gene expression either in cis (on neighboring genes) or in trans (on distant genes). Some of them are able to modulate the activity of chromatin modifiers. A paradigmatic example for this class of lncRNAs is HOTAIR, which is required for Polycomb Repressive Complex 2 (PRC2) occupancy and histone H3 lysine-27 trimethylation of the HOXD locus [49]. Other nuclear lncRNAs act as antisense transcripts or as decoy for splicing factors [50,51]. Cytoplasmic lncRNAs may conversely function as endogenous “sponges” for miRNAs, thus releasing miRNA repression on target genes [52,53].

Here we will review recent findings that point to regulatory non-coding RNAs as important players in the maintenance of ESCs pluripotency and cell fate choices during differentiation. We will also discuss the roles played by ncRNAs during somatic cells reprogramming.

## 2. MicroRNAs in Embryonic Stem Cells

**Figure 1:**
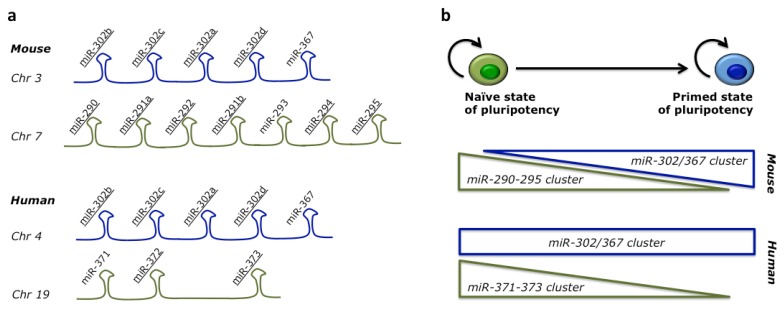
Clusters of miRNAs specifically expressed in embryonic stem cells (ESCs). (**a**) Schematic representations of the two ESC-specific miRNA clusters in mouse and human. miRNAs with the AAGUGC seed sequence within each cluster are underlined. The miR-302/367 cluster is highly conserved in mammals, whereas the other cluster is less conserved; (**b**) The two clusters are differentially expressed in ESCs during the conversion between the naïve and primed states of pluripotency.

Among all miRNAs expressed in embryonic stem cells, a single family of miRNAs with the AAGUGC seed sequence is the most highly expressed and has the most functional impact. Members of this family are organized in two major clusters (Figure 1a). The conserved miR-302/367 cluster comprises four AAGUGC seed-containing miRNAs (miR-302a, miR-302b, miR-302c and miR-302d) and the unrelated miR-367. The second cluster is less conserved. In the mouse it is commonly referred to as the miR-290-295 cluster and includes six miRNAs with the AAGUGC seed (miR-290, miR-291a, miR-291b, miR-292, miR-294 and miR-295) and miR-293. The human orthologue comprises miR-371 and the AAGUGC seed-containing miR-372 and miR-373 (miR-371-373 cluster). Therefore, multiple members with the AAGUGC seed are present in each cluster, as well as other miRNAs with a different seed [25]. For simplicity, in this review we will refer to miRNAs containing the AAGUGC seed as miR-302 family.

The miR-302 family miRNAs are abundantly expressed in undifferentiated ESCs and decline upon differentiation [54–58]. According to the miRNA expression atlas [59], miR-302 family members are specifically expressed in embryonic cells in both mouse and human. In mouse, high-throughput sequencing indicated that miRNAs belonging to the miR-290-295 cluster represent almost one third of all miRNAs expressed in undifferentiated mESCs [60]. Similarly, undifferentiated human ESCs are dominated by the mir-302 cluster, which accounts for more than 60% of all expressed miRNAs [61]. miRNAs with the AAGUGC seed are also conserved in other vertebrates, such as Zebrafish, Xenopus and Chicken, where they seem to be specifically expressed during early embryonic development [25].

The specific expression of miR-302 family miRNAs is ensured by their regulation, at the transcriptional level, by the core ESC transcriptional regulatory circuitry [8,9,62,63]. The promoters of the miR-302 and the miR-290-295 clusters are bound by Oct4, Nanog, Sox2 and Tcf3, that also promote transcription of other unrelated miRNAs. The same transcription factors, in cooperation with Polycomb group proteins, repress transcription of lineage-specific miRNAs, such as the neural miRNAs miR-9 and miR-124 and the mesodermal miRNA miR-155 [9].

Interestingly, the conversion from a naïve to a primed state in mESCs correlates with a switch between the miR-290-295 and the miR-302/367 clusters in terms of miRNA abundance [64] (Figure 1b). mESCs express high levels of miR-290-295 that decline after conversion to EpiSCs and are replaced by an increase of miR-302/367. The total levels of miRNAs with the AAGUGC seed are maintained. Since miRNAs with the same seed should share the same targets, the significance of this switch remains obscure. In human ESCs, which correspond to a primed state of pluripotency, the levels of miR-302/367 are much higher than the levels of miR-371-373 and the switch to an earlier developmental state led to an increase of the levels of miR-371-373 [65–67].

### 2.1. miRNAs Regulate ESC Cell Cycle

Inactivation of genes involved in the processing of miRNAs represents a useful approach to study the global function of miRNAs. Dicer −/− pre-gastrulation embryos showed lack of Oct4-positive epiblast cells and could not undergo gastrulation [68], indicating that the miRNA pathway is essential for establishing the pool of pluripotent embryonic cells. As expected, mESCs could not be derived from Dicer mutant embryos. Surprisingly, Dicer −/− mESCs obtained by conditional gene targeting are viable, hold an appropriate morphology, and express normal levels of pluripotency markers [69]. However, the miRNA pathway is crucial for mESC differentiation both *in vitro* and *in vivo*, as assessed by defects in lineage markers expression and teratoma formation. Moreover, inactivation of Dicer impairs the proliferation potential of mESCs [70]. As Dicer is involved in the processing of both miRNAs and other endogenous small RNAs, the observed phenotype could be due to the loss of both classes of Dicer targets. However, comparative analysis of Dicer +/+ and Dicer −/− mESCs by high-throughput sequencing indicated that very few short non-miRNA transcripts that are dependent on Dicer exist in these cells [60]. Moreover, a similar phenotype, albeit less severe, was observed in DGCR8 −/− mESCs [71], indicating that the defects detected in Dicer −/− mESCs are mainly due to the loss of miRNAs. A comparative transcriptome analysis revealed that in cells lacking all miRNAs there was a significant increase of transcripts harboring a GCACUU motif in the 3′UTR [72]. This sequence is complementary to the AAGUGC seed of miR-302 family miRNAs, thus confirming their prominent role in pluripotent stem cells. Accordingly, miR-302 family members could rescue the proliferation defects of DGCR8 mutant mESCs [73]. For their role in the regulation of the characteristic cell cycle of ESCs, these miRNAs have been called “ES-cell-specific cell-cycle-regulating” (ESCC) miRNAs [74]. Specifically, they target the Cyclin Dependent Kinase (CDK) inhibitors p21 (Cdkn1a), Rbl2 and Lats2, which are present at low levels in wt mESCs and overexpressed upon disruption of the miRNA pathway [72,73,75]. Other miRNAs may contribute to the silencing of cell cycle inhibitors in mESCs. For example, miR-320 and miR-702 target p21 and p57 [76]. Interestingly, these are non-canonical miRNAs, which require Dicer, but not DGCR8, for their biogenesis [77]. The activity of such non-canonical miRNAs might explain the more severe phenotype observed in mESCs upon loss of Dicer as compared to loss of DGCR8 [76].

Similar to mESCs, also hESCs are characterized by a shortened cell cycle, accumulating in the S phase at the expense of the G1 phase [78]. Regulation of embryonic stem cell cycle by miRNAs is evolutionary conserved, as knockdown of Dicer or Drosha impairs human ESCs proliferation [79]. This defect could be partially rescued by miR-302 family members and the unrelated miR-195. These miRNAs play distinct roles in regulating cell cycle progression. miR-195 facilitates the G2/M transition by targeting the WEE1 kinase, a negative regulator of the Cycline B/CDK complex. The miR-302 family targets p21 and promotes G1/S transition [79,80]. Another miRNA, miR-92b, participates in the regulation of the G1/S transition by targeting the CDK inhibitor p57 (Cdkn1c) [81]. Moreover, at least other two components of the cell cycle machinery, Cyclins D1 and D2, are under the control of miR-302 family members in hESCs [63,82]. Therefore, the miRNA pathway plays a major role in maintaining the peculiar cell cycle of pluripotent embryonic cells.

### 2.2. miRNAs Regulate ESC Pluripotency and Differentiation

A genome-wide approach has been recently performed in hESCs to systematically identify bona fide miRNA targets [61]. PAR-CLIP (photoactivatable ribonucleoside-enhanced cross-linking and immunoprecipitation) [83] for the RISC component AGO2, combined with perturbation of miRNA levels, led to the identification of a set of 146 high-confidence miR-302-367 direct targets. These included previously validated targets, as well as novel genes regulated by this cluster. This gene set is enriched in factors involved in the maintenance of pluripotency, such as regulators of cell cycle and proliferation, chromatin modification, metabolism and signaling [84].

**Figure 2:**
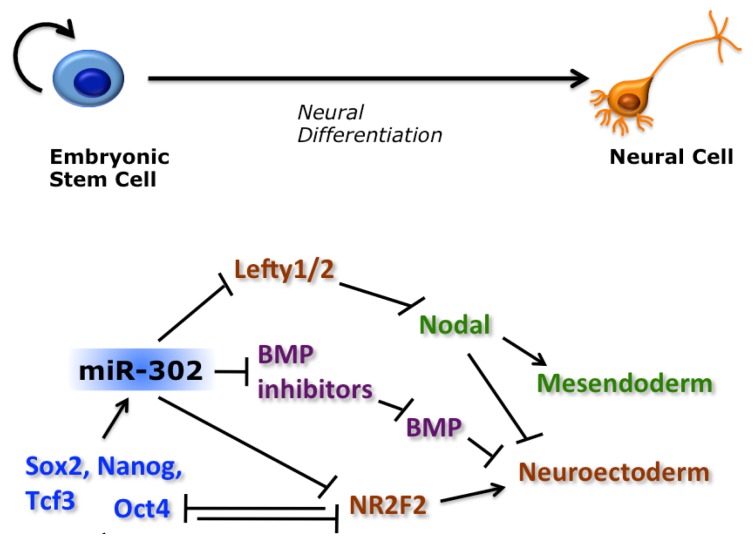
Role of miR-302 during human ESCs (hESC) neural differentiation. Transcription from the miR-302 locus is activated by the ESC core transcriptional regulatory circuitry. miR-302 post-transcriptionally inhibits NR2F2 which in turn is an inhibitor of Oct4. In a negative feedback loop, Oct4 inhibits NR2F2 transcription. NR2F2 expression is necessary for proper activation of neuroectoderm genes. miR-302 also targets inhibitors of both branches of the TGFβ pathway: the Nodal inhibitors, Lefty1 and Lefty 2, and the bone morphogenetic protein (BMP) inhibitors, DAZAP2, SLAIN1 and TOB2. According to the neural default model, inhibition of both branches of the TGFβ pathway leads to neural induction. Thus, by targeting inhibitors of both branches miR-302 has a negative effect on neural induction.

Despite holding normal morphology and expression of pluripotency markers, both mESCs and hESCs depleted of miRNAs cannot properly differentiate [69,71,79]. The miR-302 family has been involved in the TGF-β/BMP signaling pathway, which regulates embryonic stem cells pluripotency and differentiation. During hESC differentiation, Nodal activity inhibits neuroectodermal specification and promotes mesendodermal formation, whereas overexpression of the Nodal inhibitor, Lefty, results in the opposite effect [85,86]. We have shown that, by directly inhibiting Lefty, miR-302 is necessary for proper mesoderm and endoderm specification [87]. The regulation of Nodal signaling by miR-302 seems evolutionary conserved, as the Xenopus and Zebrafish hortologues target Lefty during early embryogenesis [87,88]. In addition to the inhibition of Nodal antagonists, miR-302 could promote BMP signaling by targeting the inhibitors DAZAP2, SLAIN1, and TOB2 [61]. Double blockage of the two branches of the TGF-β pathway induces a neural fate in ESCs [89,90]. By inhibiting inhibitors of both branches, miR-302 plays a central role in negatively regulating neural induction in pluripotent stem cells (Figure 2). It has recently been shown that BMP signaling down-regulates the miR-302/367 cluster in human primary pulmonary artery smooth muscle cells (PASMCs), mouse mesenchymal cells and embryonic carcinoma p19 cells [91]. In a feedback loop, miR-302 targets the type II BMP receptor (BMPRII) in PASMCs, thus inhibiting BMP signaling. Further studies are necessary to address whether this reciprocal inhibition between BMP signaling and miR-302 also exist in ESCs.

Interestingly, a regulatory loop comprising an unrelated miRNA, miR-125, and components of the BMP signaling has recently been described in mouse embryonic stem cells [92]. Such regulatory circuitry sets mESC sensitivity to BMP4. Therefore, the interplay between the TGF-β/BMP pathway and miRNAs may represent a more general regulatory mechanism modulating the response of ESC to extracellular stimuli. The interaction between miRNAs and signaling pathways underlying pluripotency might not be limited to TGF-β. In cancer cells, the Wnt/β-catenin pathway causes aberrant expression of the miR-371-373 cluster and these miRNAs, in turn, repress the activity of Wnt inhibitors, such as DKK1 [93]. It would be interesting to assess whether such interplay between the Wnt signaling and the miR-371-373 cluster has also a role in ESC pluripotency.

miR-302 is deeply integrated in the core transcriptional regulatory circuitry of ESCs. As mentioned above, the miR-302 host gene is under the control of Oct4, Nanog and Sox2, which ensure high miRNA levels in undifferentiated ESCs [9,62,63]. We have shown that both Oct4 (at the transcriptional level) and miR-302 (post-transcriptionally) repress a common target, NR2F2 (also known as COUP-TFII) [94]. NR2F2 in turn is an inhibitor of Oct4. It is activated during early neural ectoderm induction and is necessary for the proper expression of neural genes upon hESC differentiation [94]. Therefore, miR-302 and the two transcription factors, NR2F2 and Oct4, form a feedback regulatory circuitry that regulates hESC exit from pluripotency and neural fate specification (Figure 2). Individual pluripotent cell lines have different propensity to differentiate along specific lineages. Interestingly, miR-371-373 expression levels negatively correlate with the neurogenic differentiation propensity of hESC and hiPSC lines [67].

**Figure 3:**
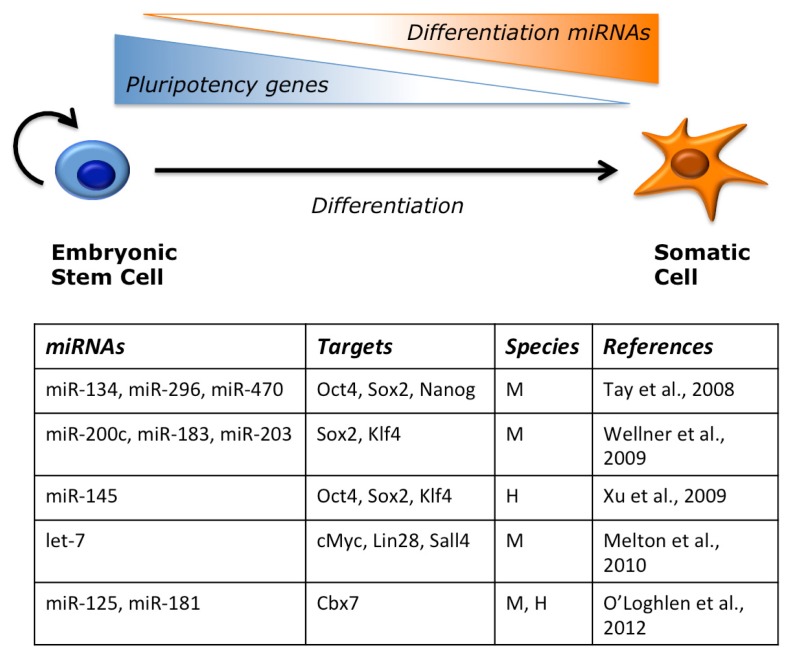
miRNAs target pluripotency genes. The activity of pluripotency genes, including core transcription factors, must be shut down as embryonic stem cells differentiate. Several miRNAs that are induced upon exit from pluripotency directly target these genes in a combinatorial way. Examples are shown in the table. M: mouse; H: human.

Epigenetic silencing of the Oct4 locus is necessary to ensure proper differentiation upon exit from pluripotency. This is achieved by de novo DNA methylation by DNMT3 factors. It has been shown that miR-290-295 play a role in this process by inhibiting Rbl2, that in turn is an inhibitor of DNMT3 [72]. In the absence of miR-290-295 there would be incomplete silencing of the Oct4 locus during differentiation. This would in part explain the differentiation defects observed in Dicer −/− cells, which maintain high levels of pluripotency factors and fail to activate lineage specific gene programs [69]. Other miRNAs may contribute to this phenotype, inhibiting the expression of pluripotency genes during mESC differentiation (Figure 3). For instance, miR-134, miR-296 and miR-470 target the core transcription factor trio, Oct4, Sox2 and Nanog [95]. Sox2 and Klf4 are also repressed by miR-200c, miR-183 and miR-203 [96]. It is interesting to notice that miR-203 has also a role in skin stem cell terminal differentiation by inhibiting p63 [97]. Pluripotency factors are under the control of miRNAs also in human ESC. An interesting regulatory loop has been shown for miR-145, which targets Oct4, Sox2 and Klf4 during differentiation, and in turn is repressed at the transcriptional level by Oct4 in undifferentiated cells [98]. Recently, it has been proposed that another role for miRNAs during ESC differentiation is to modulate the activity of chromatin modifiers. An interesting switch between different variants of the Polycomb Repressive Complex 1 (PRC1) has been shown during ESC differentiation. In particular, an ES-specific PRC1, containing the Cbx7 subunit, is replaced by a differentiation-specific PRC1, containing the subunits Cbx2/4/8 [99,100]. This switch is crucial, as Cbx proteins confer distinct target selectivity to the PRC1 complex. The different Cbx variants inhibit each other. In pluripotency conditions, Cbx7 negatively regulates the other Cbx that are conversely induced during differentiation, and repress Cbx7 transcription. miRNAs of the miR-125 and miR-181 families contribute to the downregulation of Cbx7 during mESC differentiation by directly targeting its 3′UTR [100]. Overexpression of these miRNAs in mESCs caused loss of pluripotency markers and increased expression of a subset of PRC1 target genes involved in lineage specification.

**Figure 4:**
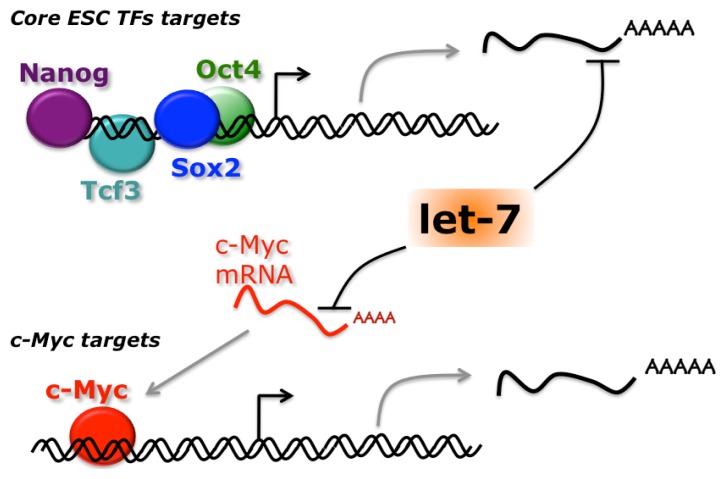
let-7 antagonizes pluripotency networks. Let-7 antagonizes indirectly the activity of the ESC core transcriptional regulatory circuitry by targeting multiple genes induced by the core transcription factors (TFs). Moreover, let-7 directly inhibits c-Myc, reducing transcription of its target genes.

Among other miRNAs with a role in ESC differentiation, the let-7 family plays a prominent role. This is a highly conserved family with orthologues in all metazoa. In mammals it comprises several let-7 species (let-7a to let-7i) and other miRNAs, such as miR-98 and miR-202 [101]. Members of the let-7 family are induced during development and differentiation, with a parallel reduction of their targets, and have a role in cancer [102]. In ESCs, levels of let-7 miRNAs are regulated at the post-transcriptional level. Whereas the primary transcript and the hairpin precursor accumulate in these cells, the production of the mature miRNA is blocked [103]. The underlying molecular mechanism relies on the recognition of the terminal loop of let-7 precursors by the RNA binding protein Lin28 [104–106]. Lin28 recruits the terminal uridylyl transferase TUT4, which in turn adds a poly-U tail, targeting the miRNA for degradation [107,108]. A feedback regulatory loop exists, in which let-7 negatively regulates Lin28 [109]. Lin28 is highly expressed in undifferentiated ESCs and declines during differentiation, when levels of mature let-7 increase. This switch is ensured by their mutual repression. As mentioned above, DGCR8 −/− ESCs maintain high levels of pluripotency genes expression. Upon transfection of let-7 family miRNAs, expression of Oct4, Sox2 and Nanog is inhibited, suggesting an anti-pluripotency activity for let-7. However, if miR-302 family miRNAs are co-transfected, they impair this activity and restore the levels of pluripotency markers [110]. Comparative transcriptome analysis showed that let-7 introduction in DGCR8 −/− mESCs led to the inhibition of genes that are transcriptionally activated by Myc factors. Both c-Myc and N-Myc are direct targets of let-7 [110]. Myc genes play a well-established role in ESC self-renewal [111]. Moreover, let-7 directly targets transcripts that are induced by other pluripotency factors, Oct4, Sox2, Nanog and Tcf3 [110]. Inhibition of the function of pluripotency factors, direct for Myc and indirect for other factors, explains the anti-pluripotency activity of let-7 (Figure 4). Conversely, Myc-activated genes and c-Myc itself are enriched among transcript that are upregulated in presence of miR-302 family members, suggesting that the pro-pluripotency activity of these miRNAs may be mediated, at least in part, by the indirect increase of Myc [110].

**Figure 5:**
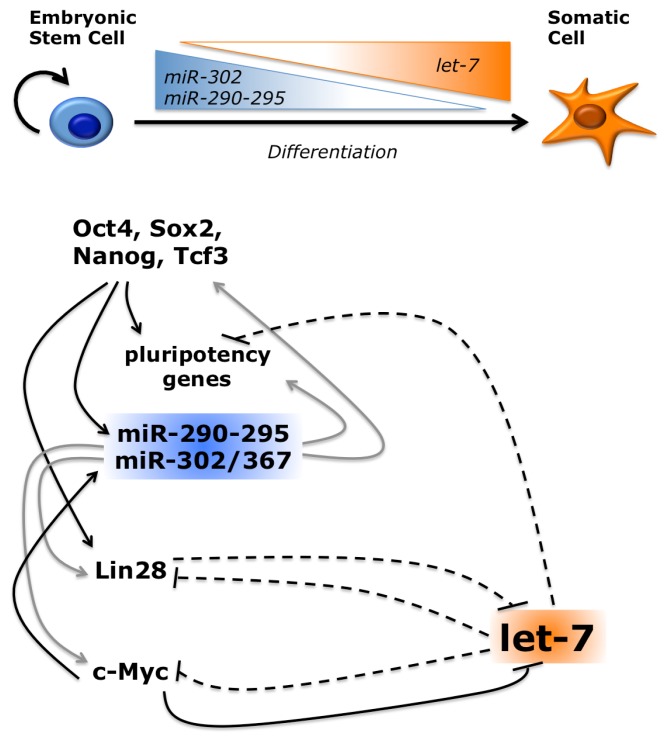
Opposing roles for the let-7 and miR-302 families. During ESC differentiation, the levels of pluripotency miRNAs belonging to the miR-302 family are decreased. In parallel, let-7 family members are induced. The miR-302/367 and miR-290-295 clusters are induced by core ESC TFs, and in a positive feedback loop indirectly promote their expression and transcription of their targets. Conversely, let-7 is post-transcriptionally inhibited by Lin-28. In cancer cells c-Myc inhibits let-7 at the transcriptional level. In a negative feedback loop, let-7 targets its inhibitors. Black arrows indicate direct activation; grey arrows indicate indirect activation; dashed lines indicate post-transcriptional inhibition.

For both let-7 and the miR-302 families feedback loops with Myc are in play. The promoter of the miR-290-295 cluster is directly bound and activated by c-Myc and N-Myc [7], and the miR-302 cluster is also induced by Myc [112], establishing positive feedback loops. Conversely, in cancer cells c-Myc has been shown to bind the promoters and repress transcription of several let-7 genes [113]. Other pluripotency factors that are directly inhibited by let-7, and indirectly activated by miR-302 family, are Lin28 and Sall4, suggesting that these miRNAs exert their function via multiple pathways [110]. Therefore, according to the model depicted in Figure 5, the miR-302 and let-7 miRNA families play opposite, crucial roles in regulating ESC pluripotency and differentiation.

## 3. MicroRNAs and Reprogramming to iPSCs

ESCs and iPSCs express a similar signature group of miRNAs, including the miR-302 family, with small differences between the two cell types [114–116]. When genetically identical mouse ESCs and iPSCs were analyzed, these differences were circumscribed to miRNAs encoded in an imprinted locus on chromosome 12qF1 [117]. Imprinted loci are transcribed in a parental specific manner and contain clusters of protein-coding and noncoding genes [118]. The 12qF1 locus includes several maternally expressed genes, including two miRNA clusters, that are aberrantly silenced in many, but not all, iPSC lines [117]. Interestingly, iPSC lines in which this imprinted locus was silenced were unable to generate all-iPSC mice upon tetraploid blastocyst complementation, due to arrest of embryonic development around mid-gestation. Conversely, iPSC lines with normal expression of the imprinted locus generated viable all-iPSC mice. Therefore, the transcriptional status of imprinted genes in the 12qF1 locus, including miRNAs, marks the development potential of different iPSC clones

### 3.1. Several miRNAs Promote or Inhibit Reprogramming

**Figure 6:**
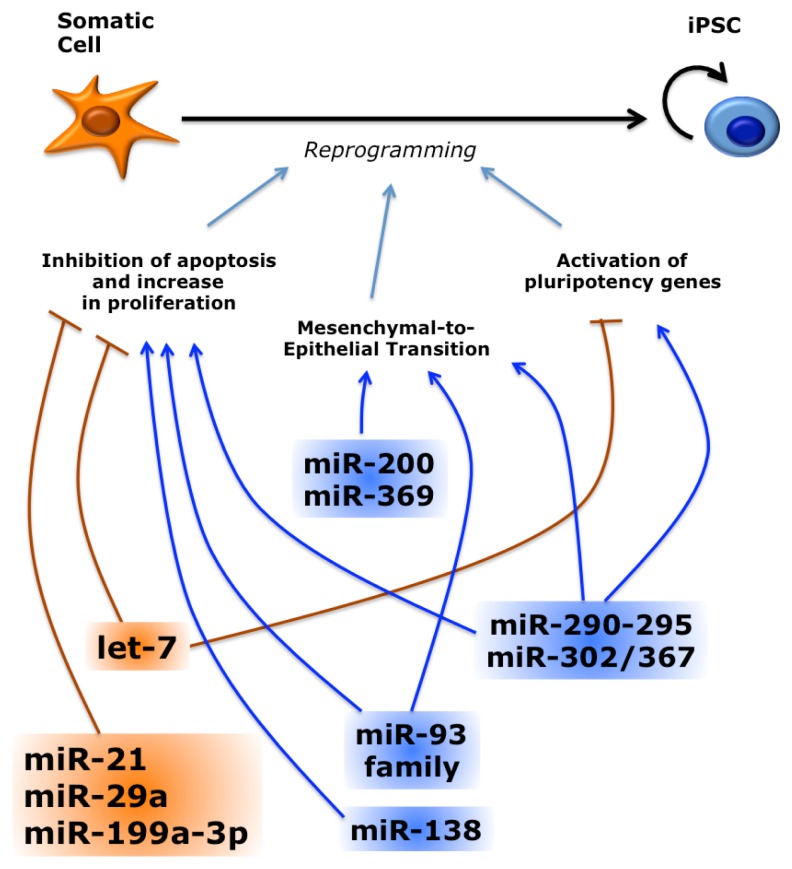
Role of miRNAs during reprogramming. The reprogramming process can be divided in three phases. Early events include inhibition of apoptosis and an increase in proliferation. A mesenchymal-to-epithelial transition (MET) occurs in the intermediate phase. Activation of the endogenous pluripotency program occurs as a late event. Several miRNAs have been shown to promote (blue) or inhibit (orange) reprogramming by facilitating or hampering the completion of these events. The miR-93 family includes miR-93 and miR-106b.

Recently, several miRNAs have been directly involved in the reprogramming process (Figure 6). Knockdown of RISC components globally impairs miRNA activity and leads to a dramatic decrease of reprogramming activity [119]. Much work concerned the miRNAs belonging to the miR-302 family. When the minimal Oct4/Sox2/Klf4 (OSK) cocktail was provided to mouse embryonic fibroblasts (MEFs) in combination with these miRNAs, the efficiency of iPSCs generation was substantially increased [119,120]. Interestingly, in presence of the three factors plus c-Myc the reprogramming enhancement by the miR-302 family members was strongly reduced [120,121]. Other miRNAs may also promote reprogramming in the mouse. For instance, miR-93 and 106b (that belong to the same family and share 5/6 of the miR-302 seed) and the unrelated miR-138 enhance iPSC generation [118,122]. Similar to the mouse system, reprogramming of human fibroblasts was also enhanced when miR-302 family members are provided along with the reprogramming factors [123].

As expected, let-7 has an opposite role during reprogramming. MEFs express high levels of let-7 and this anti-pluripotency miRNA family must be shut down during conversion into iPSCs. In the presence of let-7 inhibitors, reprogramming by the OSK cocktail was enhanced [110]. When OSK plus c-Myc were used, this increase of efficiency by let-7 inhibition was much less pronounced. Again, let-7 inhibits c-Myc, so the inhibition of let-7 might just increase c-Myc activity. Lin28, which is a component of the Thomson reprogramming cocktail and is induced during reprogramming with the Yamanaka factors, is another target of let-7. As in the case of c-Myc, addition of Lin28 to the reprogramming cocktail accelerates proliferation [124]. However, there is not an increase in MEF proliferation upon let-7 inhibition [110], suggesting that other targets besides c-Myc and Lin28 contribute to the effects of let-7 on the reprogramming process. Besides let-7, other miRNAs negatively affect reprogramming. For instance, inhibition of miR-21, miR-29a and miR-199a-3p enhances reprogramming [125,126].

### 3.2. miRNAs Reprogram Somatic Cells in the Absence of Protein-Coding Reprogramming Factors

Given their multiple roles in ESCs, the increase of reprogramming efficiency by miRNAs in combination with reprogramming factors was not unexpected. More surprisingly, recent work has shown that cocktails of miRNAs, without canonical reprogramming factors, can be sufficient to reprogram both mouse and human somatic cells. The Morrisey lab demonstrated that the miR-302-367 cluster induced pluripotency with two orders of magnitude more efficiency than standard methods [127]. Importantly iPSCs generated with miR-302-367 fulfilled stringent criteria that define bona fide pluripotent stem cells, such as the ability to contribute to chimeras with germ line transmission (mouse iPSCs) and to form teratomas (human iPSCs). In these experiments, Valproic Acid (VPA)-mediated inhibition of Hdac2 was required for mouse, but not human, miRNA-mediated reprogramming. The miR-302-367 cluster contains four miR-302 members, with the AAGUGC seed, and the unrelated miR-367. Both were required for reprogramming, as miR-302 alone is not able to give rise to iPSCs in the absence of miR-367. Interestingly, when used in combination with reprogramming factors to enhance reprogramming, miR-302 alone was almost as effective as the intact miR-302-367 cluster, whereas miR-367 alone had no effect [121]. Moreover, miR-367, but not miR-302, can be substituted by other miRNAs in alternative reprogramming cocktails. For instance, it has been shown that the combination of miR-302, miR-200c and miR-369 can reprogram both human and mouse somatic cells [128]. In this case, instead of delivering miRNA genes via viral vectors, repeated transfections of mature synthetic miRNAs were used. This approach reduces the reprogramming efficiency but might be useful towards therapeutic applications of iPSCs, which would require cells devoid of exogenous reprogramming genes integration in the genome [18]. In contrast to the finding that combinations of multiple miRNA families are necessary for reprogramming [123,127,128], Lin *et al.* have reported that miR-302 alone could convert skin cancer and hair follicle cells into iPSCs in the absence of other miRNAs or RFs [129,130]. It remains unclear whether other somatic cell types can be reprogrammed under these conditions.

### 3.3. miRNAs Regulate Reprogramming by Multiple Pathways

The mechanisms underlying miRNA-mediated reprogramming have been only partially clarified. As mentioned before, introduction of miR-302 family members in DGCR8 −/− mESCs increases expression of endogenous c-Myc and N-Myc downstream genes [110]. However, it is unlikely that the pro-reprogramming activity of the miR-302 is merely mediated by downstream activation of c-Myc. In fact, the mechanisms by which these miRNAs and c-Myc enhance the reprogramming efficiency seem different, since the miRNAs, unlike c-Myc, do not accelerate doubling time of MEFs and produce a more homogenous population of fully reprogrammed cells [120]. Multiple pathways may be affected by overexpression of these miRNAs. For instance, it has been proposed that during reprogramming miR-302 regulates multiple genes involved in cell cycle regulation, epigenetic regulation, vesicular transport, cell signaling and mesenchymal-to-epithelial transition [123].

Other miRNAs that affect reprogramming are integrated in the p53 pathway. p53 activation elicits apoptosis and cell cycle arrest, representing a roadblock to reprogramming. Consistently, p53 inhibition promotes reprogramming [131]. The anti-reprogramming miR-21 and miR-29a sustain p53 activity. As in the case of let-7, c-Myc negatively regulates these miRNAs [125]. p53 positively regulates several anti-reprogramming miRNAs: it promotes transcription of miR-34a and miR-145, which target pluripotency genes [132], and upregulates at the post-transcriptional level miR-199a-3p that inhibits cell proliferation [126]. Conversely, miR-138 enhances reprogramming by directly targeting p53 [119,122].

A mesenchymal-to-epithelial transition (MET) is one of the early events occurring during fibroblasts reprogramming to iPSCs [133,134]. Since iPSCs have an epithelial character, MET is necessary for reprogramming of mesenchymal cells, such as fibroblasts, but not for keratinocytes and other somatic cells with an epithelial character. MET is under the control of the TGFβ signaling and several reprogramming factors and miRNAs are involved in this process [135]. In particular, TGFβ promotes expression of Snail, a mediator of epithelial-to-mesenchymal transition (EMT), whereas Sox2 and Oct4 inhibit Snail transcription. c-Myc downregulates TGFβ1 and TGFβ receptor 2 (TGFBR2), which is also a target of the miR-302 and miR-93 families [119,121,123,133]. Conversely, BMP signaling stimulates expression of the miR-200 family and miR-205, which repress other EMT factors, ZEB1 and SIP1 (ZEB2) [133,134,136]. In turn, ZEB1 can inhibit transcription of the miR-200 family in a negative feedback loop [137,138]. ZEB2 is also a predicted target of miR-369 that, together with miR-200c, is a component of a miRNA-only reprogramming cocktail [128]. Another gene involved in EMT, RHOC, is a direct target of miR-302 [123].

miR-302 overexpression sustains pluripotency markers in differentiating hESCs [87,139]. Similarly, during reprogramming, miR-302 may also indirectly promote the activation of endogenous core pluripotency genes by targeting their inhibitors. For instance, methyl-DNA binding domain protein 2 (MBD2), an epigenetic suppressor of Nanog, is a direct target of miR-302 [140]. Downregulation of MDB2 by miR-302 is necessary to achieve a fully reprogrammed iPSC state. We have previously demonstrated that miR-302 targets NR2F2, which in turn is a transcriptional inhibitor of Oct4 [94]. Recently, it has been shown that NR2F2 knockdown enhances reprogramming efficiency, thus mimicking miR-302 overexpression [141].

## 4. Long Non-Coding RNAs in Embryonic Stem Cells

A number of differentially expressed lncRNAs were detected in undifferentiated mESCs and upon induction of differentiation by microarray analysis [37]. They could be classified as pluripotency, early mesoderm, and hematopoietic lncRNAs. New intergenic transcripts can be predicted by taking advantage of chromatin immunoprecipitation followed by deep sequencing (ChIP-Seq). Genes actively transcribed by Pol II are characterized by a distinctive chromatin signature, consisting in trimethylation of lysine 4 of histone H3 in the promoter combined with trimethylation of lysine 36 of histone H3 in the transcribed region (K4–K36 domain). A K4–K36 domain located outside of known protein-coding loci would predict a putative novel long intergenic ncRNA. In mESCs and somatic cells, this approach led to the identification of over a thousand of novel lincRNAs that were further validated by microarray and northern blot [142]. Bioinformatics analysis showed that both the promoters and the transcribed sequences of these novel lincRNAs are conserved in mammals, suggesting that they may have biological functions. More recently, the number of mESC lincRNAs has been further expanded by taking advantage of a computational method that allows the reconstruction of the whole transcriptome (Scripture) from massive cDNA sequencing (RNA-Seq) data [38]. Overall, lincRNAs expressed in ESC show high evolutionary conservation and tissue specificity, and their expression levels are generally comparable to protein coding genes. Interestingly, Scripture identified hundreds of noncoding RNAs that are partially overlapped with protein-coding genes but transcribed in the opposite orientation. Such antisense transcripts are less conserved than long intergenic ncRNAs and likely represent a distinct class [38]. Analysis of intergenic K4–K36 domains indicated that lincRNAs are present also in human ESCs [47].

Many mESC lincRNA genes are regulated by core transcription factors that bind directly their promoters [142]. In particular, about 10% of Nanog and Oct4 binding sites are associated with lincRNA genes [143]. Knockdown of individual core TFs affects the expression of about 60% of lincRNAs [144]. As expected, mESC lincRNAs levels decrease upon differentiation, as the levels of mESC TFs decline. Also for some human ESC lincRNAs there is evidence for a direct regulation by core TFs [145,146].

### lncRNAs Maintain Pluripotency in ESC

A biological role for mESC lincRNAs, initially postulated on the basis of their evolutionary conservation, has been recently experimentally demonstrated. RNAi against the lncRNA AK028326 (also known as Gomafu/Miat) resulted in morphological differentiation, reduced proliferation, decreased levels of Oct4 and other pluripotency markers and increase of trophoblast markers [143], suggesting that this lncRNA has a role in maintaining mESC pluripotency. AK028326 is activated by Oct4, establishing a positive feedback loop. RNAi against another mESC lncRNA, AK141205, also resulted in a decrease of Oct4, but in this case Nanog levels, morphology and proliferation were unaffected and differentiation genes were not induced [143]. Regulation of Oct4 by lncRNAs is conserved in human. Detailed analysis of transcripts generated by the Oct4 locus and Oct4 pseudogenes loci in human breast adenocarcinoma cells (MCF-7) has shown the existence of two transcripts, which were antisense to Oct4 (asOct4) and Oct4 pseudogene 5 (asOct4-pg5) [147]. It has been proposed that asOct4-pg5 is a functional lncRNA in MCF-7 cells, as it is able to recruit chromatin modifiers to the Oct4 promoter, leading to transcriptional silencing of the Oct4 gene by methylation of H3K27 and H3K9 by Ezh2 and G9a, respectively. It would be interesting to assess weather this regulatory mechanism has also a role in human pluripotent stem cells, which rely on Oct4 for the maintenance of pluripotency. Recent work has shown that three lncRNAs, lncRNA_ES1, lncRNA_ES2 and lncRNA_ES3, were specifically expressed in undifferentiated hESCs, and absent in somatic tissues [145]. For two of them, direct regulation by Oct4 and Nanog has been proposed. Functional analysis showed that the three ES lncRNAs are necessary to maintain pluripotency in hESCs, as their individual knockdown by RNAi resulted in downregulation of pluripotency markers and induction of lineage genes. Nuclear localization and association with PRC2 and Sox2 suggest that these lncRNAs may function by regulating gene expression at the transcriptional level [145].

Large-scale analysis suggests that the role of lncRNAs in pluripotent stem cells may be greater than expected. Loss-of-function by RNA interference was systematically achieved for 147 mESC lincRNAs [144]. Strikingly, for nearly all lincRNAs tested (93%), this produced a significant impact on global gene expression. On average, the number of genes affected by lincRNA knockdown was comparable to that obtained by the knockdown of previously characterized key ESC regulatory factors, suggesting that lincRNAs play major roles in mESCs. This was confirmed by further analysis, showing that knockdown of a subset of lincRNAs affected expression of pluripotency markers, including Oct4, Sox2 and Nanog, and led to a change in mESC morphology. Moreover, loss of function of many lincRNAs triggered the activation of specific neuroectoderm, endoderm, mesoderm, or trophectoderm genes [144]. These results indicate that lincRNAs are necessary for two crucial aspects of mESC pluripotency: the maintenance of the specific genetic program associated with the undifferentiated state, and the repression of genes involved in lineage differentiation.

Mechanistically, mESC lncRNAs may exert their function by modulating the activity of chromatin-modifying factors. Both “readers”, “writers” and “erasers” histone modifiers and the chromatin-associated DNA binding protein Yy1 have been found in association with mESCs lncRNAs [37,144]. For example, the lncRNAs Evx1as and Hoxb5/6as, which are transcribed antisense to the homeotic genes Evx1 and Hoxb5–Hoxb6, are associated with the Trithorax protein MLL1. Interestingly, many lincRNAs are bound by multiple chromatin regulatory complexes, which form consistent combinations of readers, writers and erasers. This evidence suggests that in mESCs lincRNAs may function as a bridge to tether distinct functionally related complexes, as proposed in other systems [148]. Such molecular scaffolding function may play a crucial role in maintaining the epigenetic state of pluripotent stem cells.

## 5. Long Non-Coding RNAs in Induced Pluripotent Stem Cells

**Figure 7:**
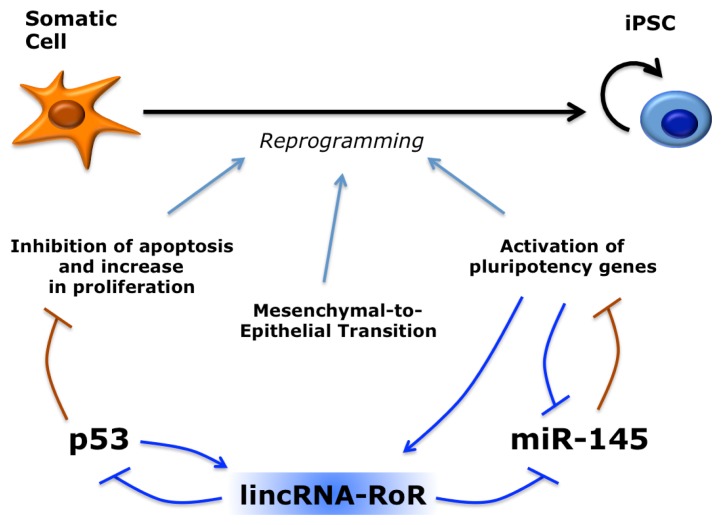
Role of lincRNA-RoR during reprogramming. LincRNA-RoR may promote reprogramming by two different mechanisms. It inhibits p53, which in turn negatively affects the initial steps of reprogramming by inducing apoptosis. It also acts as a competing endogenous RNA and releases from repression the endogenous pluripotency genes Oct4, Sox2 and Klf4, which are targeted by miR-145. MiR-145 is also inhibited by Oct4 in a negative feedback loop. Other miRNA may be “sponged” by lincRNA-RoR (not shown).

A subset of human lincRNAs demonstrated expression at higher levels in iPSCs with respect to both somatic cells of origin and hESCs [146]. Among them, there is at least one with a role in reprogramming, named lincRNA-RoR (or linc-RoR) for “Regulator of Reprogramming” (Figure 7). Knockdown of linc-RoR resulted in a significant decrease of iPSC colonies formation, whereas overexpression enhanced reprogramming efficiency. This pro-reprogramming effect can be explained by recent work showing that linc-RoR is a negative regulator of p53 [149]. Mechanistically, linc-RoR inhibits translation of p53 mRNA in the cytoplasm and this inhibitory activity is dependent on the interaction with heterogeneous nuclear ribonucleoprotein I (hnRNP I). Interestingly, linc-RoR transcription is induced by p53, establishing an autoregulatory feedback loop [149]. Another function recently proposed for linc-RoR may underlie its pro-reprogramming activity [150]. By sequestering a set of miRNAs that target Oct4, Nanog and Sox2, Linc-RoR may protect pluripotency factors from miRNA inhibition by acting as a competing endogenous RNA (ceRNA) [52].

## 6. Conclusions and Future Perspectives

In ESCs and iPSCs, pluripotency is the result of: (i) the interplay between multiple signaling pathways; (ii) a core transcriptional regulatory circuitry; (iii) epigenetic control of gene expression by specific chromatin modification patterns; (iv) the maintenance of a peculiar cell cycle. Recent work suggests that regulatory ncRNAs have a role in modulating all these biological pathways. Similar to the core set of transcription factors, ESCs are characterized by a core set of miRNAs, belonging to the miR-302 family. These miRNAs are hierarchically on top of multiple targets, which participate in the maintenance of self-renewal, and regulate exit from pluripotency and cell fate choice. Moreover, miR-302 family members are required for efficient reprogramming of somatic cells and, in combination with other miRNAs, are sufficient for iPSC generation in the absence of canonical reprogramming factors. A similar role has been shown for other miRNAs in different systems. For instance, neural-specific miRNAs can induce trans-differentiation of fibroblasts into induced neurons without other factors [151]. Since miRNAs can be provided as mature RNA molecules, this approach could lead to the generation of therapeutic relevant cells devoid of exogenous genes integrations and genomic lesions.

The functions of lncRNAs in pluripotent cells are less characterized. So far, large datasets generated by RNA-seq projects have been followed only by a limited number of functional studies. Such are hampered by the fact that unlike protein-coding genes and miRNA the function of a lncRNA cannot be normally predicted by the analysis of its sequence [152]. However, the few examples in which the function of lncRNAs has been investigated suggest that they might be primary regulators of pluripotency, differentiation and reprogramming. In particular, since lncRNAs have been show to act as molecular scaffolds of chromatin regulators, they may have a huge impact in the context of stem cells where the maintenance of a characteristic epigenetic state of the chromatin is central for pluripotency and differentiation. So far, ESC lncRNAs have been associated with maintenance of pluripotency factors, repression of differentiation, and inhibition of apoptosis. Their possible role in other crucial pathways underlying pluripotency has not yet been investigated. For instance, it is unknown whether lncRNAs could be involved in tuning signaling pathways, whereas evidence exists for miRNAs. Finally, as cytoplasmic lncRNAs may work as inhibitors of miRNAs, they could add another layer of complexity to the regulatory circuitries underlying pluripotency.
